# Mitogenomes Reveal Alternative Initiation Codons and Lineage-Specific Gene Order Conservation in Echinoderms

**DOI:** 10.1093/molbev/msaa262

**Published:** 2020-10-07

**Authors:** Zheng Bin Randolph Quek, Jia Jin Marc Chang, Yin Cheong Aden Ip, Yong Kit Samuel Chan, Danwei Huang

**Affiliations:** 1 Department of Biological Sciences, National University of Singapore, Singapore, Singapore; 2 Tropical Marine Science Institute, National University of Singapore, Singapore, Singapore

**Keywords:** mitochondrial phylogeny, Asteroidea, mitochondrial gene evolution, mitochondrial gene order, Echinodermata, translation table

## Abstract

The mitochondrial genetic code is much more varied than the standard genetic code. The invertebrate mitochondrial code, for instance, comprises six initiation codons, including five alternative start codons. However, only two initiation codons are known in the echinoderm and flatworm mitochondrial code, the canonical ATG and alternative GTG. Here, we analyzed 23 Asteroidea mitogenomes, including ten newly sequenced species and unambiguously identified at least two other start codons, ATT and ATC, both of which also initiate translation of mitochondrial genes in other invertebrates. These findings underscore the diversity of the genetic code and expand upon the suite of initiation codons among echinoderms to avoid erroneous annotations. Our analyses have also uncovered the remarkable conservation of gene order among asteroids, echinoids, and holothuroids, with only an interchange between two gene positions in asteroids over ∼500 Ma of echinoderm evolution.

In 1968, Crick proposed that although the overall genetic code is likely to be conserved through deep evolutionary time, translation initiation codons may differ among taxa (Crick 1968). Deviations from the standard code were first discovered in the human mitochondrion (Barrell et al. 1979), with a multitude of code variations that have since been uncovered (Lobanov et al. 2010). A key difference between the standard and alternative codes is in the initiation codon, for which the canonical ATG is now known to be just one of numerous possibilities. For example, up to 47 possible start codons have been found in the model bacterium *Escherichia coli* (Hecht et al. 2017).

The mitochondrial genes of echinoderms are translated according to a variant genetic code (translation table 9) distinct from the standard code. Based on a 3,849-bp fragment of mitochondrial DNA from *Asterina pectinifera* (Echinodermata: Asteroidea), Himeno et al. (1987) uncovered a number of variations in the genetic code, such as AGA and AGG coding for serine instead of arginine, and hypothesized that ATT and ATA could be alternative initiation codons for the NADH dehydrogenase subunit 3 (ND3) and NADH dehydrogenase subunit 5 (ND5) genes, respectively (Asakawa et al. 1995). Apart from the standard ATG, the only accepted alternative mitochondrial initiation codon for echinoderms is GTG (Jacobs et al. 1988; Cantatore et al. 1989). The aforementioned studies have suggested ATA, ATC, and ATT as possible start codons, although there have been reservations (Cantatore et al. 1989). To date, the validity of initiation codons in the echinoderm mitochondrial code other than ATG and GTG remains uncertain. Conversely, the mitochondrial genetic code of other invertebrates (translation table 5) comprises five alternative initiation codons—the two present in the echinoderm mitochondrial code, along with ATC, ATT, and TTG. Interestingly, ATT is the most frequently annotated start codon for ND3 among invertebrates in an investigation of over 900 mitogenomes, although ATG remains the dominant initiation codon for all protein-coding genes (PCGs) in translation tables 5 and 9 (Donath et al. 2019). The same study also found that multiple other initiation codons have been annotated for echinoderms, including all alternative start codons in translation table 5 plus TTT, TAT, GAT, and CTG.

In this study, we assembled eight new complete and three incomplete mitogenomes across ten species of asteroid sea stars for analysis with 16 published mitogenomes. For two genes, ND3 and NADH dehydrogenase subunit 4L (ND4L), we found unambiguous evidence for several species initiating with either ATT (Asteroidea only; ND3, *n *=* *16; and ND4L, *n *=* *12) or ATC (ND4L only; Asteroidea, *n *=* *7; and Echinoidea, *n *=* *3) (Fig. 1), but no taxa had mitochondrial initiation codon ATA (Materials and Methods and see Cantatore et al. 1989). We furthermore found preliminary evidence in *Euretaster insignis* for TTG being a fourth alternative initiation codon through reannotation of the NADH dehydrogenase subunit 1 (ND1) to be in frame with the other mitogenomes. TTG was also the initiation codon for ND3 (as annotated by MITOS2; Bernt et al. 2013b). Indeed, inspection of our ND3 alignment revealed that *E. insignis* had one less codon at the 5′ end than other echinoderm species. The codon in ND3 that preceded TTG, AAT, is not a known mitochondrial initiation codon in echinoderms or even invertebrates more generally, although it is a possible start codon in *Escherichia coli* (Hecht et al. 2017). Nevertheless, TTG is an established alternative initiation codon in invertebrate mitochondria (Okimoto et al. 1990), so it possibly also initiates translation of ND1 and ND3 in *E. insignis* and other echinoderms (see Donath et al. 2019). More mitogenomes from its close relatives (order Velatida) would clarify this relationship with other asteroids and the components of the mitochondrial genetic code.

**Fig. 1.**
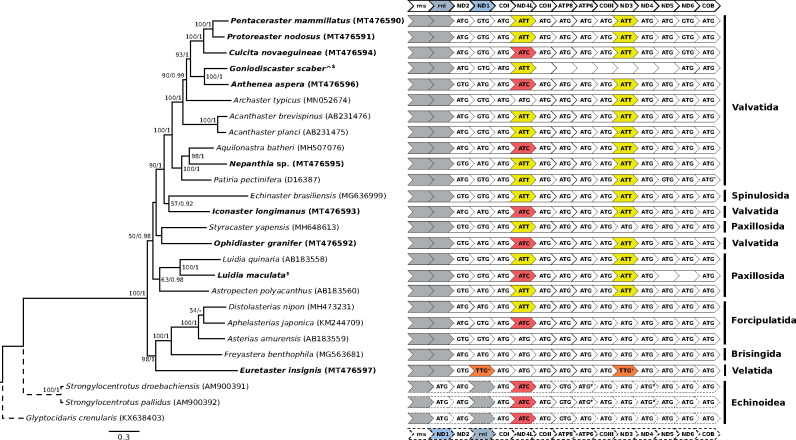
Maximum likelihood phylogeny of Asteroidea mitochondrial genomes (solid lines) with Echinoidea as outgroup (dotted lines) (left) and mitochondrial initiation codons arranged in gene order (right). Values on nodes represent maximum likelihood bootstrap (≥50)/posterior probability (≥0.90) values. Samples sequenced in this study are in bold. ^Two *Goniodiscaster scaber* samples pooled into a single terminal. *^/#^Annotated as ATT and ATA respectively in GenBank but corrected to ATG (see main text for details). ^+^Possible novel initiation codon but inconclusive (see main text for details). ^$^See Data availability.

Considering the deep divergence between Asteroidea and Echinoidea in the Cambrian (O’Hara et al. 2014), the conservation of mitochondrial gene order within each of these taxa is remarkable (Fig. 1). Examination of 21 asteroid, 26 echinoid, and ten holothuroid mitogenomes revealed that there has only been an interchange in ND1 and 16S rRNA gene positions, and both echinoids and holothuroids share the same gene order (Fig. 2). These echinoderm taxa have a relatively stable order of mitochondrial genes despite diverging from one another as far back as ∼500 Ma (O’Hara et al. 2014). In contrast, clades that have diversified over similar geological time (e.g., Ophiuroidea [*n *=* *3] and Crinoidea [*n *=* *3] [Perseke et al. 2008], as well as Corallimorpharia [Lin et al. 2014; Quattrini et al. 2020]) display much greater variability in gene arrangements (Fig. 2).

**Fig. 2.**
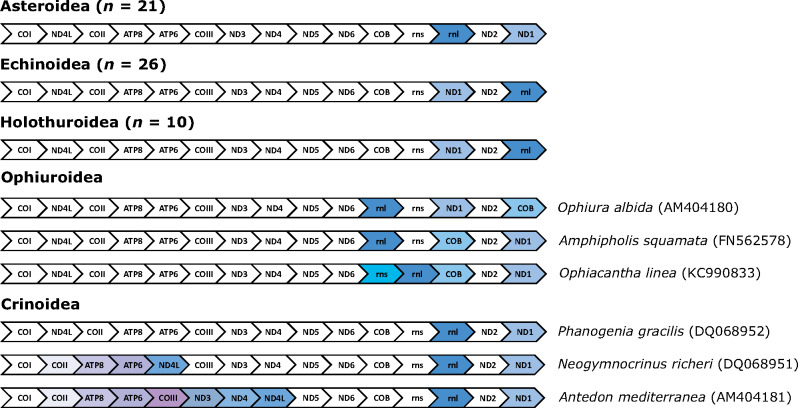
Mitochondrial gene order among the five living classes of Echinodermata.

In particular, the remaining echinoderm classes, Ophiuroidea and Crinoidea, exhibit larger variations in gene order compared with their relatives in Echinodermata. Lee et al. (2019) examined 15 species of ophiuroid mitogenomes and found limited gene order conservation compared with asteroids, echinoids, and holothuroids, although the block of genes between cytochrome c oxidase subunit I (COI) and NADH dehydrogenase subunit 6 (ND6) were ordered identically among the four classes (see Scouras et al. 2004). Based on 17 mitogenomes, Galaska et al. (2019) found similar patterns and estimated that rearrangements of rRNA genes occurred ∼175–205 Ma for two ophiuroid species (*Ophiacantha linea* and *Amphipholis squamata*; see also O’Hara et al. 2017). Mitochondrial gene order in Crinoidea is most varied, with some crinoids (e.g., *Antedon mediterranea* and *Neogymnocrinus richeri*) no longer showing the conserved block of genes present in the other four echinoderm classes. In general, nucleotide substitution rate is positively linked to gene order variation (Shao et al. 2003; Xu et al. 2006; Bernt et al. 2013a; see also Yokobori et al. 2004, 2005). Therefore, the more rapid mitochondrial nucleotide substitution in ophiuroids and crinoids (Scouras et al. 2004) could be driving the differences seen among echinoderm classes.

Although targeted sequencing of fast-evolving mitochondrial markers remains useful for phylogenetic inference (e.g., mitochondrial control region, Bronstein et al. 2018), complete mitochondrial genes and genomes are now readily obtainable via whole-genome sequencing (e.g., Galaska et al. 2019; this study), and even as by-products of restriction site-associated DNA (Terraneo et al. 2018a, 2018b) or hybrid-capture (Allio et al. 2020; Quek et al. 2020) sequencing. Mitogenome sequences are frequently used in downstream applications such as population genomics and phylogenomics (Quek et al. 2019; Barrett et al. 2020; Inoue et al. 2020; Poliseno et al. 2020). Apart from homologous gene sequences isolated from mitogenomes, gene order can also be useful for elucidating phylogenetic relationships, particularly for ancient divergences as mitochondrial DNA sequences are likely to be substitution saturated due to the rapid evolutionary rates (Boore and Brown 1998; Oxusoff et al. 2018). For example, Fritzsch et al. (2006) reconstructed the phylogeny of protostomes based on mitochondrial gene arrangements and recovered the monophyly of arthropods, annelids, platyhelminths, and nematodes. However, such methods are more applicable for deep relationships across a broad range of taxa (Boore and Brown 1998; Lavrov and Lang 2005; Fritzsch et al. 2006), or among species with a number of gene rearrangements (Chen et al. 2018; but see Tyagi et al. 2020).

The mitogenome phylogeny reconstructed here (Fig. 1) is consistent with the phylotranscriptomic analysis of Linchangco et al. (2017) in showing that Velatida, Brisingida, and Forcipulatida share a close relationship and are sister to Valvatida, Spinulosida, and Paxillosida. The paraphyly of Valvatida has been reported in previous studies (Janies et al. 2011; Mah and Blake 2012) and also noted by Linchangco et al. (2017). The latter recovered Paxillosida as a clade, though we note that Ophidiasteridae (represented here by *Ophidiaster granifer*) was not analyzed. Although Ophidiasteridae is conventionally placed within Valvatida, Mah and Blake (2012) have reported that Ophidiasteridae is more closely related to Paxillosida. Nevertheless, we note that the sister relationship between *Styracaster yapensis* (Paxillosida) and *Ophidiaster granifer* as well as the general polyphyly of Paxillosida is not well supported by both reconstructions (Fig. 1). Resolution of these relationships and revision of the Asteroidea classification are warranted but require broader species and gene sampling.

Overall, this study has highlighted not just the diversity of the genetic code and the conserved gene order of many echinoderm mitogenomes but also improved gene annotations arising from the accurate identification of initiation codons.

## Materials and Methods

New mitogenomes sequenced in this study consisted of 11 taxa, of which two were previously sequenced together with *Archaster typicus* in Quek et al. (2019) and the remaining nine samples were from Ip et al. (2019) and the cryogenic collection of the Lee Kong Chian Natural History Museum (for sample information, see http://dx.doi.org/10.5281/zenodo.3834170). DNA extraction, library preparation, sequencing, read quality trimming, and assembly followed by mitogenome contig identification and annotation largely followed Quek et al. (2019), but sequencing was performed on a HiSeq 4000 (150×150 bp). Raw reads were trimmed using Trimmomatic v0.38 (Bolger et al. 2014) and assembled with SPAdes v3.12.0 (Bankevich et al. 2012) under default settings. A BlastN (*e*-value = 10e^−6^) was conducted against two *Acanthaster* mitogenomes (Yasuda et al. 2006) to identify mitochondrial contigs, which were then annotated using MITOS2 (Bernt et al. 2013b).

Data obtained were combined with published, annotated mitochondrial sequences for an additional 16 species from GenBank. From the 27 mitogenomes, 13 PCGs and both rRNAs were extracted and aligned. Annotations of some PCGs by MITOS2 (Bernt et al. 2013b) had discrepancies in lengths when aligned to reference mitogenomes downloaded from GenBank, so all PCGs were aligned and annotations adjusted following visual inspection to ensure that they were in frame and accurately annotated.

Cytochrome b, ATPase subunit 6, and NADH dehydrogenase subunit 4 in three published mitochondrial genomes were originally annotated with alternative initiation codons ATA (*Strongylocentrotus droebachiensis* [AM900391] and *S. pallidus* [AM900392]) or ATT (*Patiria pectinifera* [D16387]), but inspection of gene alignments allowed the start codon to be corrected as the standard ATG codon (Fig. 1). Sequences from three samples could not be assembled into complete mitochondrial genomes (Fig. 1), so only full-length genes were extracted for phylogenetic analysis.

Sequence matrix preparation and phylogenetic analyses were conducted as described in Quek et al. (2019) with phylogenetic reconstruction performed based on maximum likelihood implemented in RAxML v8.2.11 (Stamatakis 2014) and Bayesian inference carried out in MrBayes v3.2.6 (Ronquist et al. 2012).
